# Association of TNF-α gene with spontaneous deep intracerebral hemorrhage in the Taiwan population: a case control study

**DOI:** 10.1186/1471-2377-10-41

**Published:** 2010-06-10

**Authors:** Yi-Chun Chen, Fen-Ju Hu, Phoebe Chen, Yih-Ru Wu, Hsiu-Chuan Wu, Sien-Tsong Chen, Guey-Jen Lee-Chen, Chiung-Mei Chen

**Affiliations:** 1 Department of Neurology, Chang Gung Memorial Hospital Linkou Medical Center and College of Medicine, Chang-Gung University, Taoyuan, Taiwan; 2 Department of Life Science, National Taiwan Normal University, Taipei, Taiwan; 3 Department of Agricultural Chemistry, National Taiwan University, Taipei, Taiwan

## Abstract

**Background:**

Genetic factors may play a role in susceptibility to spontaneous deep intracerebral hemorrhage (SDICH). Previous studies have shown that *TNF-α *gene variation was associated with risks of subarachnoid hemorrhage in multiple ethnicities. The present case-control study tested the hypothesis that genetic variations of the *TNF-α *gene may affect the risk of Taiwanese SDICH. We examined the association of SDICH risks with four single nucleotide polymorphisms (SNPs) within the *TNF-α *gene promoter, namely T-1031C, C-863A, C-857T, and G-308A.

**Methods:**

Genotyping was determined by PCR-based restriction and electrophoresis assay for 260 SDICH patients and 368 controls. Associations were tested by logistic regression or general linear models with adjusting for multiple covariables in each gender group, and then in combined. Multiplicative terms of gender and each of the four SNPs were applied to detect the interaction effects on SDICH risks. To account for the multiple testing, permutation testing of 1,000 replicates was performed for empirical estimates.

**Results:**

In an additive model, SDICH risks were positively associated with the minor alleles -1031C and -308A in men (OR = 1.9, 95% CI 1.1 to 3.4, p = 0.03 and OR = 2.6, 95% CI 1.3 to 5.3, p = 0.005, respectively) but inversely associated with -863A in females (OR = 0.5, 95% CI 0.2 to 0.9, p = 0.03). There were significant interaction effects between gender and SNP on SDICH risks regarding SNPs T-1031C, C-863A, and G-308A (p = 0.005, 0.005, and 0.007, respectively). Hemorrhage size was inversely associated with -857T in males (p = 0.04).

**Conclusions:**

In the Taiwan population, the associations of genetic variations in the *TNF-α *gene promoter with SDICH risks are gender-dependent.

## Background

Deep parenchymal structure including the basal ganglia, thalamus, brainstem, and cerebellum is the most frequently (65% to 80%) affected site of spontaneous intracerebral hemorrhage (SICH) [1,2]. SICH developing at deep parenchymal structure (SDICH) varies in size and location, and thus differs in clinical presentations and prognosis according to the arteries that have ruptured. SICH is a devastating stroke subtype, in which the six-month and one-year mortality rate of SICH ranged from 23% to 58% and >50%, respectively [3,4]. The 30-day mortality rate is associated with the initial Glasgow Coma Scale and hemorrhage size [5]. In addition, males tended to have a higher risk (more than twice), larger hemorrhage size, and a poorer outcome than females [3,6]. We have shown sex differences in the etiological spectrum of SDICH in the young, in which cryptogenic etiology and underlying vascular anomaly are significantly higher in females while hypertension attributes to a higher SDICH risk in males [6].

A recent article comprehensively reviewed the candidate genes of SICH reported during 1996 to 2007 [7]. Candidate genes reported as associated with SICH were involved in the pathways of the vessel wall integrity (*ACE, APOE, neprilysin, endoglin, TGF-β1*), endothelial dysfunction (*ACE*), inflammation markers (*IL-6, TNF*), and hemostasis (*APOE, CD-14, Factor VII and XIII, VKORC1*). However, these studies were mainly conducted in lobar SICH. In contrast, the number of genetic studies of SDICH is limited [2,8-10].

As a complex disease, SDICH aggregate in families but does not segregate in a mendelian fashion [2,6,11]. Family aggregation in SDICH group suggests involvement of shared genetic and/or environmental factors on SDICH risks [2,10]. To our knowledge, susceptibility genes of SDICH remain largely unknown. Although hypertension is the most important risk factor for SDICH [3,12], it accounts for only 54% of SDICH cases [2]. Genetic predisposition has been speculated to account for the novel pathophysiology, including hemostatic pathway, pathways involving endoglin and cytochrome P450 [13].

Concentrations of inflammation markers in plasma and cerebral spinal fluid have been shown to be positively correlated with mortality in SICH patients [14]. Tumor necrosis factor *α *(TNF-*α*) is one of the main proinflammatory cytokines and plays a central role in initiating and regulating the inflammatory response. The *TNF-α *gene is located in 6p21.3. Previous studies have shown that polymorphisms at position -308 and -863 in the promoter region of the *TNF-α *gene were associated with risks of subarachnoid hemorrhage (SAH) in multiple ethnicities [15,16]. Differences in transcriptional activator resulting in different TNF-*α *concentrations may contribute to this association [17,18]. In contrast to the finding in SAH, there is no evidence showing an association of *TNF-α *polymorphism with SICH in a Japanese study [16]. To our knowledge, there is no report addressing the association of *TNF-α *polymorphism with SDICH risk.

The purpose of this study is to evaluate whether any particular polymorphism of *TNF-α *gene would predispose to SDICH and modify the 30-day outcome of SDICH events in the Taiwan population.

## Methods

### Subjects

This is a retrospective case-control study. Subjects were recruited from the Department of Neurology, Chang Gung Memorial Hospital (CGMH), Linkou Medical Center, which is a tertiary referral center serving residents mainly in the northern Taiwan. Patients with SDICH were diagnosed based on both clinical presentations and brain computed tomography (CT) [3,10,19]. Clinical presentations included a nontraumatic, abrupt onset of severe headaches, focal neurologic deficit, or altered consciousness level related to hematoma at the brain parenchyma that was not due to hemorrhagic transformation of a cerebral infarct. CT of brain is the first-choice of imaging options to identify the presence of acute intracerebral hemorrhage and its size and location [19]. Inclusion criteria for SDICH cases were (i) patients with SDICH and (ii) a patient or her/his legally acceptable representative, willing to provide written informed consent to participate. Patients with a SAH, traumatic hemorrhage, brain tumor, vascular anomaly, abnormal platelet count, prolonged prothrombin time, and prolonged activated partial thromboplastin time were excluded. Participants of the control group were recruited randomly from the community of the Northern Taiwan and met the following inclusion criteria: (i) subjects providing informed consent, (ii) subjects who came to CGMH for a health examination or treatment for diseases other than neurodegenerative diseases, inflammatory diseases, or cerebrovascular diseases (CVDs), (iii) no medical history of neurodegenerative diseases, inflammatory diseases, and CVDs, and (iv) all the medical records were reviewed by at least two neurologists. Approximate 90% of the controls were enrolled from subjects who visited for health examination. This study was approved by the Institutional Review Board of CGMH.

### Clinical information

Anthropometric data and 12-hour fasting blood were collected from all participants. For SDICH cases, blood was drawn within one week of event in average. Diabetes mellitus (DM) was defined based on World Health Organization (WHO) criteria. Hypertension was diagnosed when blood pressure (BP) repeatedly exceeded 140 mm Hg (systolic) and/or 90 mm Hg (diastolic) or when a subject was taking antihypertensive medication to control hypertension. For SDICH cases without prior diagnosis of hypertension, patients were considered to have hypertension when BP measured after the acute phase of SDICH (14 days within the onset of hemorrhage) repeatedly exceeded 140 mm Hg (systolic) and/or 90 mm Hg (diastolic). Body mass index (BMI) was calculated by weight in kilograms divided by squared height in meters. Alcohol use was defined as drinking ≥210 g per week.

Hemorrhage located at the basal ganglia and thalamus was defined as supratentorium SDICH, while brainstem and cerebellum hemorrhage were defined as infratentorium SDICH. Volume of the hematoma was calculated using the so-called ABC method based on CT of brain [5]. Short term outcome was assessed using the modified Rankin scale (mRS) on day 30 after SDICH events. Subjects who were dead or dependent (mRS >3) at the 30 day follow-up were considered poor prognosis [20].

### Genotyping

Genomic DNA was isolated from peripheral leukocytes using DNA Extraction Kit (Stratagene). Genotypes for *TNF-α *T-1031C, C-863A, C-857T, and G-308A were determined by PCR-based single-strand conformation polymorphism (SSCP) and/or restriction fragment length polymorphism (RFLP) assays. The details of specific PCR conditions and detection for genotyping, including polymorphism primer pair, anneal, and enzyme were described in our previous report [21]. The amplified products were digested with the appropriate restriction enzyme (New England Biolabs, Beverly, MA) and subsequently separated on 1.8%~2.2% agarose gels. In addition, aliquots of the amplified products were mixed with an equal volume of 95% formamide buffer and subjected to SSCP and heteroduplex analyses using GeneGel Excel gels as recommended by the manufacturer (Pharmacia Biotech, Uppsala, Sweden).

Each of the four single nucleotide polymorphisms (SNPs) was checked for Hardy-Weinberg equilibrium (HWE) using the standard χ^2 ^test with significance level of 0.001 in each of the case and control groups and in the combined group. Patterns of linkage disequilibrium (LD) were evaluated using Haploview v4 [22], and haplotypes were reconstructed by PHASE 2.0 [23] based on the LD results. Haplotypes with frequency <1% were excluded from association analysis.

### Statistical analysis and power estimation

The Pearson's χ^2^-test or t-test was utilized to compare demographic data and the distributions of genotypes of *TNF-α *between controls and cases. Two-tailed P-values were derived from the χ^2^-test or Fisher's exact test. Association analyses were performed first stratified by genders, and then in combined. Multivariable logistic regression was used to analyze the phenotype-genotype associations of SDICH with *TNF-α *alleles under additive genetic models. Covariables included age, sex, BMI, and hypertension, total cholesterol level, and alcohol use [3]. Because hematoma size is affected by its location, the association between hematoma and genotype effect was analyzed only in hemorrhages that were located at supratentorium. Associations between hematoma size and each of the SNPs were examined by general linear models (GLM). To examine interaction effects between gender and each of the SNPs, the multiplicative term of genotypes and gender was included and evaluated in the same additive model as the interaction term. Permutation testing of 1,000 replicates was performed for empirical estimates as a robust alternative to standard parametric tests. Permutation testing was performed only when the preliminary P-value was <0.05.

In the present case-control study, at the 5% significance level, we had power greater than 0.8 to identify an association under an additive genetic model when the per-allele genetic effect was greater than an odds ratio of 1.5 and the disease allele frequency was greater than 0.2.

All the data analyses were performed using SAS software version 9.1.3 (SAS Institute, Cary, NC, USA).

## Results

The characteristics of the study groups are presented in Table 1. A total of 260 SDICH patients and 368 controls were included in this study. Age and gender distribution did not differ between SDICH patients and controls. The proportions of hypertension and alcohol use were significantly higher in patients than in controls. Cases had lower concentration of total cholesterol (TC) than controls. Proportions of risk factors of SDICH were slightly different between genders, in which the proportion of DM was significantly higher in the female but not in the male patients. In SDICH cases, males had a lower proportion of supratentorium hemorrhage and larger hemorrhage size than females (Table 1). There was no significant gender difference in the 30-day outcome estimated by mRS.

**Table 1:** Demographic data in patients with spontaneous deep intracerebral hemorrhage (SDICH) and controls

	Males (n = 403)			Females (n = 225)			Both genders (n = 628)	
	
	SDICH (n = 177)	Controls (n = 226)	P-Value	SDICH (n = 83)	Controls (n = 142)	P-Value	SDICH (n = 260)	P-Value
Age (years)	58.3 ± 1.0	58.7 ± 0.9	0.82	65.1 ± 1.3	62.6 ± 0.9	0.05		0.61
Hypertension (%)	91.5	32.3	<0.0001	95.2	38.7	<0.0001		<0.0001
Diabetes mellitus (%)	12.4	15.9	0.35	30.1	13.4	0.004		0.32
Alcohol use (%)	46.9	19.5	<0.0001	4.8	0	0.018		<0.0001
Body mass index (kg/m^2^)	25.2 ± 0.3	25.8 ± 0.3	0.13	24.4 ± 0.4	25.1 ± 0.4	0.31		0.08
Total cholesterol (mg/dL)	176.9 ± 2.7	191.7 ± 3.0	0.0003	188.3 ± 4.1	204.6 ± 3.7	0.003		<0.0001
Triglyceride (mg/dL)	137.9 ± 6.1	143.7 ± 6.9	0.90	143.8 ± 8.3	147.3 ± 8.0	0.80		0.77
SDICH at supratentorium	149 (84.2%)			80 (96.4%)			229 (88.1%)	0.005^a^
Hemorrhage size (cm^3^)^b^	18.0 ± 1.6			13.1 ±1.6			16.3 ±1.2	0.03^a^
modified Rankin scale > 3	49.1%			52.5%			50.2%	0.62^a^

Data are expressed as percentage or mean ± SE. Comparisons between controls and SDICH cases were analyzed by χ^2 ^test or t-test where appropriate. To convert mg/dL to mmol/L, multiply cholesterol values by 0.02586 and triglycerides by 0.011.

^a^Comparisons between genders. ^b^Average size in those with supratentorium SDICH.

In the entire cohort, distributions of all the four SNPs did not differ between SDICH cases and controls (Table 2). When stratified by genders, distributions of T-1031C and G-308A were significantly different between cases and controls in males (χ^2 ^test, p = 0.01 and p = 0.02, respectively). In contrast, the distribution of C-857T differed significantly between cases and controls in females (χ^2 ^test, p = 0.002) but not in males. Each of the four SNPs was in HWE in each of the case and control groups and in the combined group. The minor allele frequencies of all the four SNPs were ≥ 0.05, and therefore we utilized the four SNPs to evaluate LD. There was a single block structure, composed of a portion of the SNPs T-1031C and C-863A with D' estimation of 0.92.

**Table 2:** Frequencies and associations of TNF-*α *genotypes in patients with spontaneous deep intracerebral hemorrhage (SDICH) and controls

	Males (n= 403)			Females (n = 225)			Total
**SNP**	**SDICH** **(n = 177)**	**Controls** **(n = 226)**	**P-Value**, **OR (95% CI) **	**SDICH** **(n = 83)**	**Controls** **(n = 142)**	**P-Value**, **OR (95% CI) **	**P-Value**, **OR (95% CI) **

T-1031C			**P = 0.03**, 1.9 (1.1 to 3.4)			P = 0.06, 0.5 (0.3 to 1.02)	P = 0.65, 1.1 (0.7 to 1.7)
							
TT	119 (67.2)	169 (74.8)		52 (62.7)	86 (60.6)		
TC	48 (27.1)	55 (24.3)		31 (37.4)	49 (34.5)		
CC	10 (5.7)	2 (0.9)		0 (0.0)	7 (4.9)		

C-863A			P = 0.09, 1.6 (0.9 to 2.7)			**P = 0.03**, 0.5 (0.2 to 0.9)	P = 0.99, 1.0 (0.7 to 1.5)
							
CC	120 (67.8)	163 (72.1)		54 (65.1)	85 (59.9)		
CA	46 (26.0)	59 (26.1)		28 (33.7)	49 (34.5)		
AA	11 (6.2)	4 (1.8)		1 (1.2)	8 (5.6)		

C-857T			P = 0.20, 0.7 (0.4 to 1.2)			P = 0.21, 1.6 (0.8 to 3.3)	P = 0.86, 0.9 (0.6 to 1.5)
							
CC	132 (74.6)	163 (72.1)		52 (62.7)	116 (81.7)		
CT	43 (24.3)	59 (26.1)		30 (36.1)	22 (15.5)		
TT	2 (1.1)	4 (1.8)		1 (1.2)	4 (2.8)		

G-308A			**P = 0.005**, 2.6 (1.3 to 5.3)			P = 0.18, 0.6 (0.2 to 1.3)	P = 0.19, 1.4 (0.8 to 2.4)
							
GG	129 (72.9)	190 (84.1)		67 (80.7)	104 (73.2)		
GA	44 (24.9)	33 (14.6)		15 (18.1)	37 (26.1)		
AA	4 (2.3)	3 (1.3)		1 (1.2)	1 (0.7)		
							

Values are n (%). OR: Odds ratio; CI: confidence interval. Logistic regression analyses, adjusting for age, sex, BMI, hypertension, total cholesterol level, and alcohol use in an additive model. P-values in bold were empirical p-values that estimated by permutation of 1000 replicates.

When adjusting for age, sex, BMI, hypertension, TC level, and alcohol use, logistic regression showed associations of the four SNPs with SDICH risks in a different direction in each gender group. Specifically, in an additive model, SDICH risks were positively associated with the minor alleles of SNPs -1031C and -308A in men (OR = 1.9, 95% CI 1.1 to 3.4, p = 0.03 and OR = 2.6, 95% CI 1.3 to 5.3, p = 0.005, respectively) but inversely associated with -863A in women (OR = 0.5, 95% CI 0.2 to 0.9, p = 0.03). These associations were not significant in the combined cohort because of the opposite direction of associations in each gender group. Multiplicative terms of SNPs and gender evaluated in the same additive models showed significant SNP-gender interaction effects on SDICH risks regarding SNPs T-1031C, C-863A, and G-308A (p = 0.005, 0.005, and 0.007, respectively). Haplotypes constructed by the SNPs T-1031C and C-863A were not associated with the SDICH risks in each gender group and in the combined group (data not shown).

In the SDICH group, 149 in 177 (84.2%) males and 80 in 83 (96.4%) females had hemorrhage located at supratentorium (gender difference p = 0.005, χ^2 ^test) (Table 1). Hemorrhage located at supratentorium was larger in size in males (18.0 ± 1.6 cm^3^) than in females (13.1 ± 1.6 cm^3^, t-test, p = 0.03). There was no association of hemorrhage location with any of the four SNPs (p > 0.05, data not shown). Regarding hemorrhage size, only SNPs C-857T showed a borderline association in male group after adjusting age, BMI, alcohol use, and hypertension in additive model (Table 3). The hemorrhage size decreased by 6.0 ± 4.0 cm^3 ^per minor allele for the -857T variant (parameter estimates derived from GLM models, p = 0.04). No significant SNPs-gender effect on hemorrhage size was found. Haplotypes constructed by the SNPs T-1031C and C-863A were not associated with hemorrhage size in each gender and in the combined group.

**Table 3:** Associations of hemorrhage size with *TNF-α *genotypes in patients with spontaneous deep intracerebral hemorrhage (SDICH)

	Males		Females		Total
**SNP**	**Hemorrhage size (cm^3^)**	**P-Value**	**Hemorrhage size (cm^3^)**	**P-Value**	**P-Value**

T-1031C		0.57		0.23	0.27
TT	18.5 ± 2.1		12.0 ± 2.2		
TC	17.4 ± 2.3		14.8 ± 2.5		
CC	15.1 ± 5.3		-		
C-863A		0.66		0.15	0.31
CC	18.2 ± 2.0		11.9 ± 2.1		
CA	18.2 ± 2.7		15.9 ± 2.7		
AA	15.7 ± 5.3		2.0 ± 0		
C-857T		**0.04**		0.58	0.23
CC	18.9 ± 1.9		12.6 ± 2.3		
CT	15.5 ± 2.6		14.1 ± 2.4		
TT	3.5 ± 0.5		2.0 ± 0		
G-308A		0.80		0.95	0.84
GG	18.3 ± 2.0		13.2 ± 1.7		
GA	17.8 ± 2.5		12.6 ± 5.1		
AA	11.6 ± 3.9		15.0 ± 0		

GLM models that included age, sex, BMI, hypertension, TC level, and alcohol use in an additive model. Data are expressed as mean ± SE.

P-values in bold were empirical p-values that estimated by permutation of 1000 replicates.

The 30-day short term outcome including dependency or death was remarkably correlated with age (multiple logistic regression, p = 0.003) and hematoma size (p < 0.001) (data not shown in tables), but neither with any of the four SNPs nor the constructed haplotypes in the male, female, and in the combined groups. There was no SNP-gender interaction effect on short term outcome.

## Discussion

Knowledge of susceptibility genes of SDICH, a common complex disease, is largely lacking because of the study obstacles of the need of *a priori *knowledge of gene function, modest genetic effect, polygenic effect, and epistasis. The study herein shows that the common genetic variations of *TNF-α *gene are associated with SDICH risks with significant gender differences. The direction of associations between *TNF-α *gene and SDICH is opposite between genders. In an additive model, SDICH risks are positively associated with the dose of minor alleles of -1031C and -308A in men, but inversely associated with -863A in female. Multiplicative terms of analyses confirm the interaction effects between gender and each of the SNPs T-1031C, C-863A, and G-308A on SDICH risks. Hemorrhage size is inversely associated with -857T allele in males. No association between the 30-day outcome and *TNF-α *gene is found in this study. Haplotypes constructed by the SNPs T-1031C and C-863A did not reveal any association with SDICH risks, hemorrhage size, and mRS.

Increased serum TNF-*α*, one of the main proinflammatory cytokines regulating an inflammatory response, has been demonstrated to be associated with increased mortality rate of SICH [14] and recurrent stroke [24]. Previous studies have demonstrated that the G allele of the G-308A in the Italy population [15] and the A allele of the C-863A in the Japanese population [16] are associated with SAH risks. It is worth mentioning that the Italy study [15] consisted of more women (n = 115) than men (n = 56), and the analyses in their study was not stratified by gender. In contrast, there was no association between SICH risks and SNPs of C-863A, C-850T, G-238A in the Japanese population [16]. This study, however, shows associations between SDICH risks and each of the SNPs T-1031C, C-863A, and G-308A. The strength of our study to show the significant results partly comes from the homogeneous phenotype in our study, in contrast to the previous Japanese study which included both lobar SICH and SDICH [13,16]. Lobar hemorrhage and non-lobar hemorrhage are considered to be attributable to different etiologies [25]. The major cause of SDICH is hypertension [1,2,12], while lobar SICH is related to cerebral amyloid angiopathy [3]. In addition, the sample size of our study is larger than the prior one and thus had more power to identify the association between SNPs and SDICH risk. However, the present study only suggested modest genetic effects on SDICH risks.

Polymorphisms in the regulatory region of promoter may result in different TNF-*α *concentration. The SNPs -308A [17], -1031C, -863A, and -857T [18] have been shown to be associated with increasing *TNF-α *expression. Possible mechanisms of the influence may come from substances binding to regulatory elements, such as nuclear factor κB and organic cation transporter 1, and alteration of the secondary structure of DNA to affect accession of *cis*-acting transcription factors to the promoter/enhancer region of the *TNF-α *gene [26,27]. The study herein showed remarkable gender differences in the associations between promoter polymorphisms of *TNF-α *and SDICH risks. In males, we found that minor alleles of the *TNF-α *variation are risks to SDICH as hypothesized. However, the direction of the association was opposite in females. Several clinical and experimental studies have shown gender differences in the cytokine response to stress [28,29]. Type 1 lymphocytes that produce interleukin (IL)-12, IL-1β and TNF-*α *are increased in men as compared to women after experimental lymphocyte stimulation in an *ex vivo *study [28].

Our study also showed an inverse association of -857T with hemorrhage size in males. Because of the antifibrinolytic effects of TNF-*α *by stimulating the release of plasminogen activator inhibitor type 1 and by reducing the release of t-PA through the extrinsic route [30], the release of TNF-*α *may be responsible for the early activation of hemostatic mechanism during a SDICH event [31]. The -857T allele may produce a higher level of TNF-*α*, which may in turn decrease the hemorrhage size.

It is worth mentioning that patients in this study were ascertained from the Department of Neurology, which might cause a smaller average hemorrhage size than that of patients in the Department of Neurosurgery. Furthermore, this study does not have sufficient subjects of infratentorium hemorrhage to examine the correlation between genetic effects and the significant gender difference in the hemorrhage loci. Also, the number of female cases is relatively small, which is due to a lower incidence rate compared to males [32] rather than selection bias. A multiplicative term of gender and SNPs showed significant interaction effects between gender and SNPs T-1031C, C-863A, and G-308A. Further replicated study is needed to confirm the results herein, especially regarding the hemorrhage size and short term outcome. In addition, the SNPs we studied may not pose a specific risk factor for SDICH, given that these SNPs could be a risk factor for numerous conditions [18,21,27]. This report is preliminary, the effect sizes are small and the study will need to be replicated before these SNPs can be viewed as independent risk factors for SDICH. The other limitation of this study is that we did not measure serum TNF-*α *concentration, which may otherwise provide additional functional information to support our hypothesis. A further study examining the correlation between the SNPs and serum TNF-*α *concentration will be useful. Nevertheless, our study has several strengths. This study included a homogenous disease entity in a same ethnic background, which may limit the confounding effect from multiple phenotypes and ethnicities. This is the first study analyzing associations between *TNF-α *genotype and the SDICH susceptibility, and independent confirmation is needed to verify these associations.

## Conclusions

This study showed a modest effect of *TNF-α *polymorphisms on SDICH risks and size with significant gender differences.

## Competing interests

The authors declare that they have no competing interests.

## Authors' contributions

YCC contributed to study design, analysis, interpretation of data, and drafted the manuscript. FJH carried out the molecular genetic studies. PBC carried out the molecular genetic studies. YRW contributed to acquisition of data. HCW made substantial contributions to conception and design. STC participated in the design of the study and has been involved in drafting the manuscript. GJLC made substantial contributions to the molecular genetic studies. CMC contributed to study design, molecular genetic studies, interpretation of data, and has given final approval of the version to be published.

## Pre-publication history

The pre-publication history for this paper can be accessed here:

http://www.biomedcentral.com/1471-2377/10/41/prepub
